# Characterization of a heat-tolerant *Chlorella* sp. GD mutant with enhanced photosynthetic CO_2_ fixation efficiency and its implication as lactic acid fermentation feedstock

**DOI:** 10.1186/s13068-017-0905-y

**Published:** 2017-09-12

**Authors:** Tse-Min Lee, Yu-Fei Tseng, Chieh-Lun Cheng, Yi-Chuan Chen, Chih-Sheng Lin, Hsiang-Yen Su, Te-Jin Chow, Chun-Yen Chen, Jo-Shu Chang

**Affiliations:** 1 Department of Marine Biotechnology and Resources, National Sun Yat-sen University, Kaohsiung, 80424 Taiwan; 2 Doctoral Degree Program in Marine Biotechnology, National Sun Yat-sen University, Kaohsiung, 80424 Taiwan; 3 Department of Chemical Engineering, National Cheng-Kung University, Tainan, 70146 Taiwan; 4 Department of Biological Science and Technology, National Chiao Tung University, Hsinchu, 30068 Taiwan; 5 Doctoral Degree Program in Marine Biotechnology, Academia Sinica, Taipei, 11529 Taiwan; 6 Department of Biotechnology, Fooyin University, Kaohsiung, 83102 Taiwan; 7 University Center for Bioscience and Biotechnology, National Cheng-Kung University, Tainan, 70146 Taiwan

**Keywords:** *Chlorella* sp., CO_2_ utilization efficiency, Lactic acid, Light conversion efficiency, Mutagenesis, Photosynthesis, *N*-methyl-*N*′-nitro-*N*-nitrosoguanidine (MNNG)

## Abstract

**Background:**

Fermentative production of lactic acid from algae-based carbohydrates devoid of lignin has attracted great attention for its potential as a suitable alternative substrate compared to lignocellulosic biomass.

**Results:**

A *Chlorella* sp. GD mutant with enhanced thermo-tolerance was obtained by mutagenesis using *N*-methyl-*N*′-nitro-*N*-nitrosoguanidine to overcome outdoor high-temperature inhibition and it was used as a feedstock for fermentative lactic acid production. The indoor experiments showed that biomass, reducing sugar content, photosynthetic O_2_ evolution rate, photosystem II activity (*F*_v_/*F*_m_ and *F*_v_′/*F*_m_′), and chlorophyll content increased as temperature, light intensity, and CO_2_ concentration increased. The mutant showed similar DIC affinity and initial slope of photosynthetic light response curve (α) as that of the wild type but had higher dissolved inorganic carbon (DIC) utilization capacity and maximum photosynthesis rate (*P*_max_). Moreover, the PSII activity (*F*_v_′/*F*_m_′) in the mutant remained normal without acclimation process after being transferred to photobioreactor. This suggests that efficient utilization of incident high light and enhanced carbon fixation with its subsequent flux to carbohydrates accumulation in the mutant contributes to higher sugar and biomass productivity under enriched CO_2_ condition. The mutant was cultured outdoors in a photobioreactor with 6% CO_2_ aeration in hot summer season in southern Taiwan. The harvested biomass was subjected to separate hydrolysis and fermentation (SHF) for lactic acid production with carbohydrate concentration equivalent to 20 g/L glucose using the lactic acid-producing bacterium *Lactobacillus plantarum* 23. The conversion rate and yield of lactic acid were 80% and 0.43 g/g *Chlorella* biomass, respectively.

**Conclusions:**

These results demonstrated that the thermo-tolerant *Chlorella* mutant with high photosynthetic efficiency and biomass productivity under hot outdoor condition is an efficient fermentative feedstock for large-scale lactic acid production.

## Background

Increase in carbon dioxide (CO_2_) emissions derived from fossil fuel combustion is one of the main causes of global warming [1]. Microalgae are among the most promising solution for carbon sequestration and also an ideal feedstock for the production of biofuels [2]. Furthermore, microalgae are also capable of biosynthesizing high value products like pigments and omega-3 fatty acids [3]. On the other hand, using carbohydrate-rich microalgal feedstock for the fermentative production of fine chemicals (such as succinic acid, lactic acid, and so forth) has received increasing attentions [4, 5]. Among the bio-based chemicals produced from microalgae, lactic acid has been recognized with high potential for commercialization [6].

Lactic acid (2-hydroxypropanoic acid) is an important chemical because of its wide applications in the food, pharmaceutical, cosmetic, textile, and chemical industries [7, 8]. In addition, it can be used as a feedstock for the production of poly-lactic acid (PLA), an environmentally friendly biodegradable and biocompatible polymer used in surgical sutures, orthopedic implants, drug delivery systems, and disposable consumer products, which can be substituted for petrochemical plastics [9, 10]. Almost all lactic acid is now produced through fermentation of carbohydrate-rich materials [6]. Because the manufacturing cost of raw materials is still high [11], various renewable materials are considered as economic resources for efficient lactic acid production and to reduce the carbohydrate feedstock costs [6, 12]. In comparison to lignocellulosic biomass, microalgae that do not contain lignin have been considered as a suitable alternative substrate for fermentative lactic acid production. A freshwater green microalga *Hydrodictyon reticulum* that contains 47.5% reducing sugars has been used as a carbohydrate substrate for the production of l-lactic acid by *Lactobacillus* sp. isolated from the traditional Korean food, makgeolli [13, 14]. The acid hydrolysate (5% H_2_SO_4_, 120 °C, 1 h) of a marine green microalga *Nannochloropsis salina* after lipid extraction, a lipid-free algal hydrolysate with glucose and xylose as dominant reducing sugars, has also been used as a substrate for fermentative lactic acid production by *Lactobacillus pentosus* with a lactic acid yield of 0.93 g/g algal biomass at sugar concentration ranging from 3 to 25 g/L [15].

An increase in temperatures limits the growth performance of microalgae in field conditions, particularly in photobioreactors (PBRs) [16]. The water temperatures in tropical and subtropical regions, particularly in the closed PBRs, frequently surpass 40–45 °C without temperature control. The optimal growth temperature for most algae is in the range of 20–30 °C, although some species show improved heat tolerance [17–19]. Therefore, outdoor culture of microalgae generally encounters overheating issues. For example, overheating of the fed batch cultures of *Chlorella pyrenoidosa* in tubular PBR in the field resulted in a decrease of biomass when culture temperature went above 40 °C [20]. Both growth (biomass productivity) and photosynthetic performance (*F*_v_/*F*_m_) of *Chlorella sorokiniana* in outdoor cultures were reduced when temperature as well as dissolved oxygen concentrations increased [21]. Due to the possible lethality to algae as a consequence of probable overheating in closed PBR, temperature control is of major concern. In addition to applying improved engineering strategies for temperature control, thermo-tolerant algal species can be chosen to overcome high-temperature inhibition during outdoor PBR culture.

An indigenous green microalga, *Chlorella* sp. GD was isolated from piggery wastewater in Taiwan and exhibited high biomass productivity of 0.852 g/L/day in indoor PBRs using piggery wastewater as nutrient source [22], and it can be used as a feedstock for fermentative lactic acid production. Because water temperatures in outdoor PBRs frequently surpass 40 °C during the daytime in hot summer season in Taiwan, overheating of PBR becomes a problem for outdoor growth of *Chlorella* sp. GD and its subsequent application in fermentative lactic acid production. This study therefore aims to screen the thermo-tolerant *Chlorella* sp. GD mutants with high photosynthetic efficiency after random chemical mutagenesis using *N*-methyl-*N*′-nitro-*N*-nitrosoguanidine (MNNG) [23] for improving outdoor biomass production and also to investigate the suitability of algal biomass as a feedstock for fermentative production of lactic acid. We have selected one mutant with elevated biomass productivity and high CO_2_ utilization efficiency under high-temperature condition. The optimal conditions (temperature, light intensity, and CO_2_) for biomass production were examined in indoor and outdoor PBRs and the biomass was harvested as a feedstock for fermentative production of lactic acid.

## Results

### Isolation of *Chlorella* sp. GD mutant with enhanced thermo-tolerance and high CO_2_ utilization ability

**Fig. 1:**
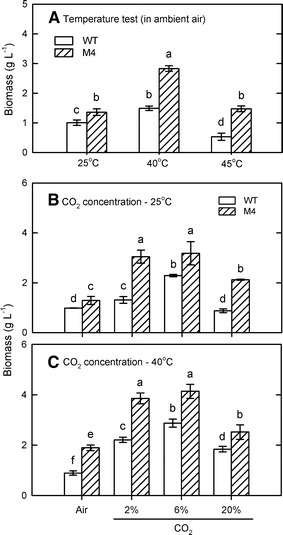
Interaction of temperature and CO_2_ concentration on algal growth in indoor PBR. The growth performance of *Chlorella* sp. GD wild type and mutant M4 was determined first under different temperatures for 4 days (**A**) and then under different CO_2_ concentrations at 25 °C (**B**) and 40 °C (**C**) for 4 days. The data are expressed as the mean ± SD (*n* = 3) and different letters indicate significant difference between the culture times (*P* < 0.05)

After MNNG mutagenesis and high-temperature (40 °C) screen, twenty-one mutants that showed higher biomass production have been isolated. Of them, one mutant (M4) that exhibited a twofold increase in biomass production after 4 days at 40 °C incubation (Fig. 1A) was chosen for further studies. The biomass production was 2.9 g/L when cultured in 75 mL BG-11 medium in a 125-mL PBR (Fig. 1A).

Next, the utilization of CO_2_ by the mutant and its interactions with temperature were studied. The biomass of both wild type and M4 increased when CO_2_ concentration was increased to 2–6% and M4 showed a higher increase in biomass with the maximum at 6% CO_2_ aeration (Fig. 1B). The biomass production of M4 can be further increased up to 4.2 g/L by 6% CO_2_ aeration under 40 °C condition.

**Fig. 2:**
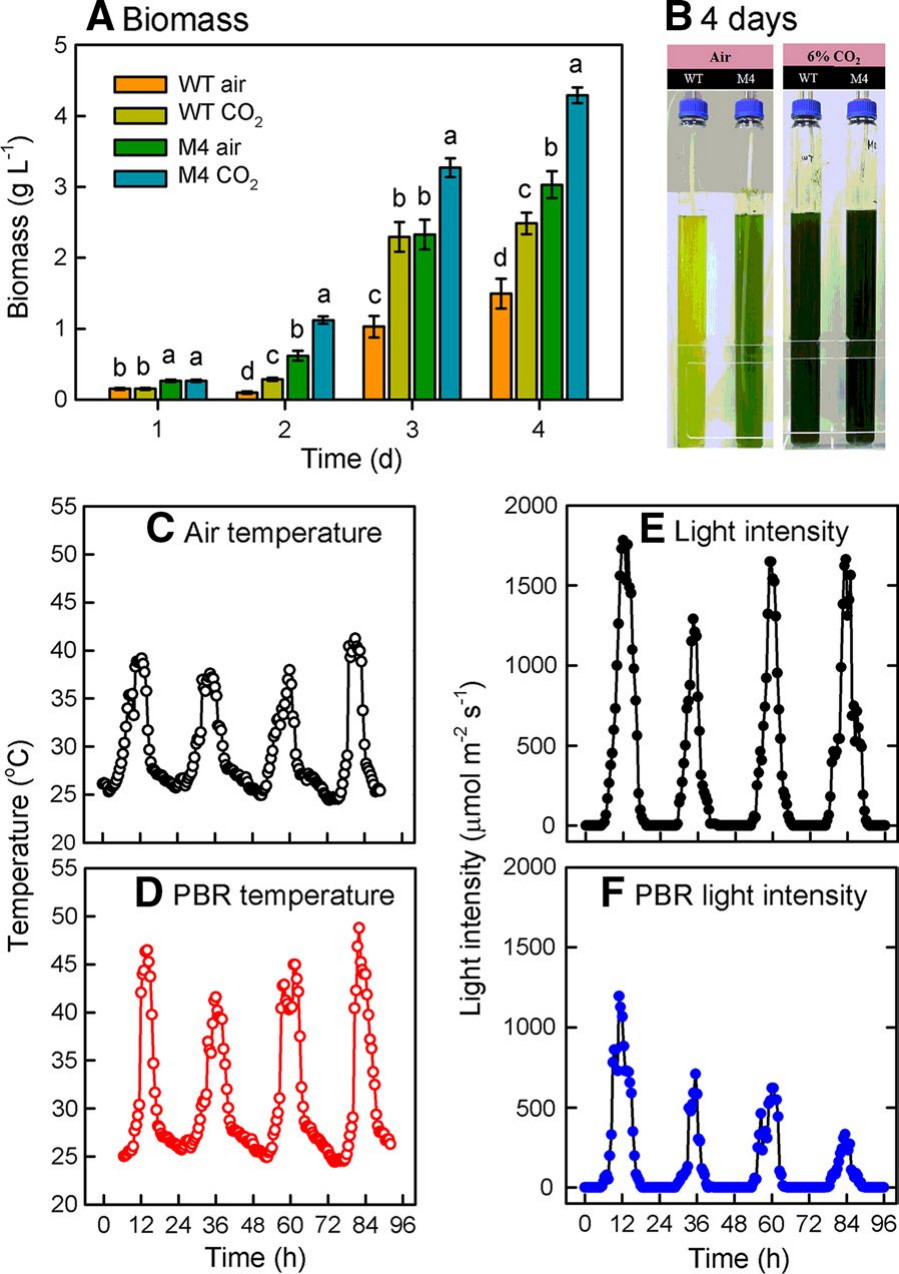
Growth performance in outdoor PBR. Biomass production (**A**) and appearance (**B**) of *Chlorella* sp. GD wild type and mutant M4 were determined in outdoor PBR aerated with 6% CO_2_. The data are expressed as the mean ± SD (*n* = 3) and different letters indicate significant difference between the culture times (*P* < 0.05). Air temperature (**C**), PBR temperature (**D**), irradiance outside PBR (**E**), and irradiance inside PBR (**F**) were recorded over culture period

Whether M4 still showed a better growth performance under hot outdoor condition was tested using a 1-L outdoor PBR set in the Aquafarm of College of Marine Sciences, National Sun Yat-Sen University, Kaohsiung, Taiwan (22°37′54.79″N, 120°15′39.82″E), from August 3 to 7, 2015. We found that although the biomass of both wild type and M4 increased with time, M4 exhibited a higher biomass production as compared to the wild type whenever it was aerated with ambient air or 6% CO_2_ (Fig. 2A). Furthermore, aeration with 6% CO_2_ resulted in a higher biomass production than air aeration, and the biomass productivity of wild type and M4 after 4 days of 6% CO_2_ aeration was 0.65 and 1.02 g/L/day, respectively (Fig. 2A). The algal cells appeared dark green for mutant M4 grown in ambient air and 6% CO_2_ and wild type grown at 6% CO_2_, but for the wild type grown with ambient air aeration, the cells were light green (Fig. 2B). During the 4 days of culture, the weather was hot and clear, as reflected by high temperature (Fig. 2C) and high light intensity (Fig. 2E). The water temperature in the PBR could increase up to 43–49 °C during daytime and was down to around 26–28 °C during the night (Fig. 2D), while the maximum light intensity in the center of the PBR was 1280 μmol/m^2^/s at day 1 and the peak light intensity on every day decreased as time increased, possibly due to increased cell density (Fig. 2F).

**Fig. 3:**
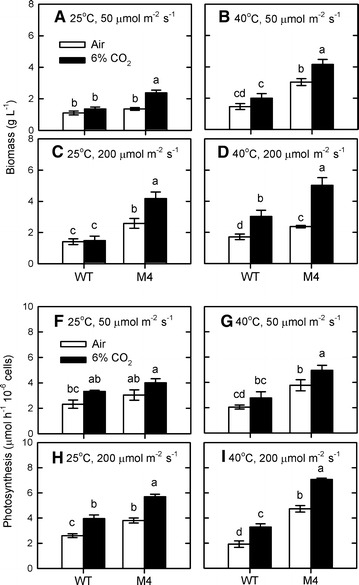
Interactive effects of temperature, light, and CO_2_ concentration on algal growth and photosynthesis. The effects of temperature (25 and 40 °C), light intensity (50 and 200 μmol/m^2^/s), and CO_2_ concentration (ambient air and 6% CO_2_) on biomass production (**A**–**D**) and photosynthetic O_2_ evolution rate (**E**–**H**) of *Chlorella* sp. GD wild type and mutant M4 were determined 4 days after culture in indoor PBR. The data are expressed as the mean ± SD (*n* = 3) and different letters indicate significant difference between the culture times (*P* < 0.05)

**Fig. 4:**
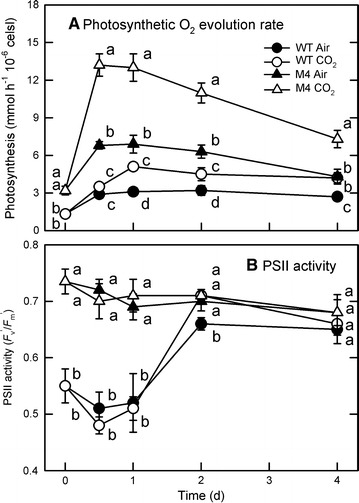
Time-course changes in photosynthetic activity in indoor PBR. The photosynthetic O_2_ evolution (**A**) and PSII activity (*F*_v_′/*F*_m_′) (**B**) of wild type and mutant M4 of *Chlorella* sp. GD were determined in indoor PBR aerated with ambient air or 6% CO_2_ at 40 °C under 200 μmol/m^2^/s illumination. The data are expressed as the mean ± SD (*n* = 3) and different letters indicate significant difference between the culture times (*P* < 0.05)

Because light intensity is known for its influence on algal growth [21], the interactive effect of light intensity (50 and 200 μmol/m^2^/s) with temperature (25 and 40 °C) and CO_2_ concentration (air and 6%) on biomass production in relation to photosynthetic efficiency was carried out in the mutant and the wild type. When light intensity increased, both biomass and photosynthetic O_2_ evolution rate of the mutant M4 were higher than those of the wild type (Fig. 3). Furthermore, both biomass (Fig. 3A–D) and photosynthetic O_2_ evolution rate (Fig. 3H–K) in the mutant can be increased by increasing temperature (from 25 to 40 °C), CO_2_ concentration (6% CO_2_), or light intensity (from 50 to 200 μmol/m^2^/s). Both biomass productivity and photosynthetic O_2_ evolution rate were maximal under the combination of 40 °C, 6% CO_2_, and 200 μmol/m^2^/s (Fig. 3). Then, the photosynthetic performance was further investigated by examining the time-course dynamics in photosynthetic O_2_ evolution rate and PSII activity under 40 °C and 200 μmol/m^2^/s condition. The data in Fig. 4 showed that the photosynthetic O_2_ evolution rate in the mutant peaked on day 1 and decreased gradually, while in the wild type a delayed response appeared with the peak after 2 days. The active PSII activity (*F*_v_′/*F*_m_′) in the wild type was around 0.55 during 0–1 days and then increased to 0.7 after 2 days, while that in the mutant it was 0.7 over the culture period (Fig. 4B). The CO_2_ aeration did not change the *F*_v_′/*F*_m_′ values in both wild type and M4 (Fig. 4B). The maximum PSII activity (*F*_v_/*F*_m_) showed a similar trend (data not shown). Our findings demonstrated that the mutant showed normal PSII activity in stock culture and after a transfer to the PBR, whereas the wild type exhibited decreased PSII activity in stock culture and the acclimation process was needed for the wild type to recover after a transfer to the PBR.

### Photosynthetic light response curve (PI curve) and dissolved inorganic carbon (DIC) utilization curve

**Fig. 5:**
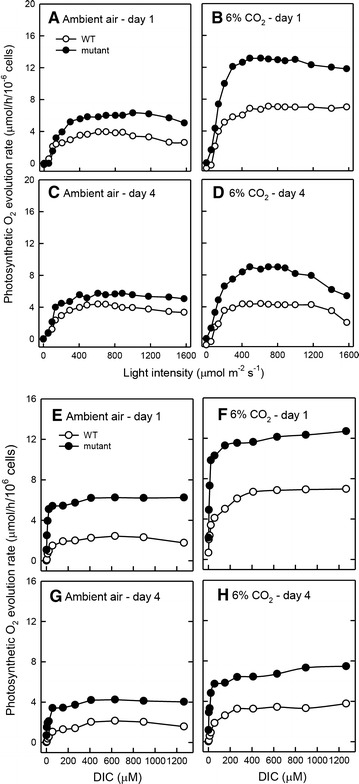
Comparison of photosynthetic light response curve (PI curve) (**A**–**D**) and dissolved inorganic carbon (DIC) utilization curve (**E**–**H**). PI curve and DIC utilization curve were determined in wild type and mutant M4 of *Chlorella* sp. GD after 1 day and 4 days of culture in indoor PBR aerated with ambient air (**A**, **C**, **E**, **G**) or 6% CO_2_ (**B**, **D**, **F**, **H**) at 40 °C under 200 μmol/m^2^/s illumination. The data are expressed as the mean ± SD (*n* = 3)

The above results have indicated that the characteristics of photosynthesis were modified in the mutant with enhanced thermo-tolerance. Thus, the photosynthetic light response curve (PI curve) was determined using algal cells grown under high light intensity condition (200 μmol/m^2^/s) to characterize its photosynthesis. As shown in Fig. 5A, the maximum photosynthesis rate (*P*_max_) was higher in the mutant during early (day 1) to late (day 4) culture period, especially when aerated with 6% CO_2_. However, the initial slope of photosynthetic light response curve (α) was similar not only between wild type and M4 but also between air aeration and CO_2_ aeration over the culture period (Fig. 5A). It reflects that the mutant exhibited a higher light utilization activity under CO_2_ aeration.

In addition to a change in light utilization efficiency, the photosynthetic kinetics with respect to dissolved inorganic carbon could be also modified. The photosynthetic O_2_ evolution rate in response to increasing dissolved inorganic carbon (DIC) concentration was assayed. The *K*_1/2_ (DIC) value was calculated to evaluate the affinity for DIC. The mutant showed a lower *K*_1/2_ (DIC) value whenever aerated with air or 6% CO_2_, and this trend was similar at day 1 and at day 4 (Fig. 5). The maximum rate of photosynthetic O_2_ evolution under saturated DIC concentration was significantly higher in the mutant and also under 6% CO_2_ condition (Fig. 5).

These results demonstrated that both light utilization efficiency and DIC utilization efficiency have been modified in this thermo-tolerant *Chlorella* sp. GD mutant.

### C and N contents, sugar compositions, and fermentative lactic acid production

To elucidate the possibility of using the biomass of *Chlorella* sp. GD mutant M4 as a fermentation feedstock for lactic acid production, the following parameters were analyzed: the C and N content (%, dry weight) and C/N molar ratio of wild type (WT) and mutant and their reducing sugar content after acid hydrolysis with 4% (v/v) H_2_SO_4_.

These tests were first performed using the cultures obtained from indoor PBR. We found that C% and N% in the mutant were higher than those in the wild type under air aeration and therefore resulted in a similar C/N ratio between wild type and M4. While the aeration with 6% CO_2_ increased C% in both wild type and M4 and decreased their N%, where C% and N% were similar between wild type and M4, leading to a similar C/N ratio between wild type and M4 under 6% CO_2_ aeration (Table 1). After H_2_SO_4_ hydrolysis, the monosaccharides present in the hydrolysate were glucose and xylose, with glucose as the dominant sugar. The glucose content was significantly higher in the mutant M4 and can be slightly increased by CO_2_ aeration (Table 1). In contrast, the xylose content was not different between wild type and mutant, but can be increased by 6% CO_2_ aeration (Table 1). The total reducing sugar content was 39.69% in the wild type and 45.02% in the mutant after 4 days of culture under 6% CO_2_ aeration.

**Table 1:** Comparison of C and N content between *Chlorella* sp. GD wild type and mutant M4

Condition	Strain	C%	N%	C/N	Reducing sugar after acid hydrolysis		
					Glucose (%)	Xylose (%)	Total sugar (%)
Air	WT	43.89 ± 0.38b	6.56 ± 0.11b	7.8 ± 0.09c	16.47 ± 0.98c	8.9 ± 0.51b	25.37 ± 1.03c
	M4	48.82 ± 0.24a	7.17 ± 0.04a	7.94 ± 0.05c	31.09 ± 1.65a	8.6 ± 0.84b	39.69 ± 3.62a
6% CO_2_	WT	49.24 ± 0.60a	5.06 ± 0.08c	11.63 ± 0.09a	23.17 ± 1.89b	11.16 ± 0.99a	34.33 ± 2.85b
	M4	50.33 ± 0.72a	5.52 ± 0.13c	10.69 ± 0.17b	34.05 ± 2.32a	10.97 ± 0.83a	45.02 ± 2.76a

C and N content (%, w/w, dry weight) and C/N molar ratio of *Chlorella* sp. GD wild type (WT) and M4 mutant after 4 days of aeration with air or 6% CO_2_ using laboratory bottle, and their reducing sugar content after acid hydrolysis. The data are presented as mean ± SD (*n* = 3) and the different letters following the SD indicate the significant difference at *P* < 0.05

**Fig. 6:**
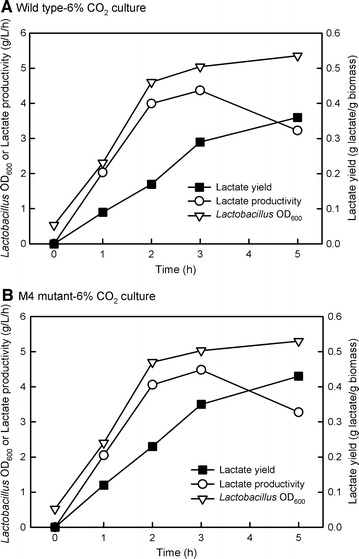
Time-course profile of lactic acid yield, lactic acid productivity, and the growth (OD_600_) of *Lactobacillus* plantarum 23 during lactic acid fermentation using microalgal biomass as feedstock. The feedstock from WT (**A**) and M4 mutant (**B**) strains was obtained from 8-day culture under aeration with 6% CO_2_. The content of glucose in wild type (WT) and M4 mutant in the fermentative mixture was adjusted to around 21 g/L, while the xylose content was around 2 g/L

Because mass culture will be carried out in outdoors for future industrial application using algal biomass as a feedstock, the sugar content in algal biomass harvested from outdoor culture was determined to check whether the mutant still showed higher sugar content under outdoor culture. The culture period was 8 days and 1 g NaNO_3_ was added at the beginning of the culture and again after 4 days of culture to prevent N deficiency during prolonged culture. After 8-day outdoor culture (September 10–18, 2015), the biomass of wild type and M4 was 6.16 g/L and 8.22 g/L, respectively, and the content of total reducing sugar, glucose, and xylose in the mutant was 59.83, 49.93, and 4.90%, respectively, and for the wild type it was 48.57, 44.54, and 4.03%, respectively (Table 2). Based on the initial biomass of 0.162 g, the biomass productivity of wild type and M4 was 0.75 and 1.01 g/L/day, respectively. The fermentation yield of acid hydrolysate was 0.36 and 0.43 g lactic acid/g biomass in wild type and mutant, respectively, with lactic acid production of 2.18 and 3.56 g lactic acid/L PBR in wild type and M4, respectively (Table 2). As shown in Table 3, the fermentation efficiency (lactic acid conversion rate) for standard glucose, wild type biomass, and M4 biomass were 74.87, 80.42, and 79.17%, respectively, with a complete conversion within 5 h. It is worth noting that the conversion efficiency using algal biomass as a feedstock was higher than that of using pure glucose. The consumption of glucose and xylose was complete at 5 h of fermentation for all samples, while the titer of lactic acid production after 5 h of fermentation was 14.50, 16.10, 16.38 g/L for standard glucose, wild type biomass, and M4 biomass, respectively (Table 3). The time-course relationship between the growth of *Lactobacillus plantarum* 23 and lactic acid production has been shown in Fig. 6. In both wild type (WT) and M4 mutant feedstock, as OD_600_ of *L.* plantarum 23 increased with time, the lactic acid productivity (g/L/h) increased to a peak at 3 h, following a decline, while the lactic acid yield (g lactate/g biomass) increased to the maximum after 5 h.

**Table 2:** Sugar compositions, fermentative lactic acid production, and conversion efficiency

Strain	Biomass	Start substrate (g)	Reducing sugar after acid hydrolysis			Lactic acid yield (g/g biomass)	Lactic acid production (g lactic acid/L PBR)
			Glucose (%)	Xylose (%)	Total sugar (%)		
WT	6.16 ± 0.32	47.15	44.54 ± 1.31	4.03 ± 0.52	48.57 ± 2.01	0.36	2.18
M4	8.22 ± 0.51*	39.28	54.93 ± 1.03*	4.90 ± 0.31*	59.83 ± 1.10*	0.43	3.56

The biomass (g dry weight/L PBR), glucose and xylose contents (%, g/g biomass) of *Chlorella* sp. GD wild type (WT) and M4 mutant after acid hydrolysis using 4% (v/v) H_2_SO_4_, as well as the lactic acid production performance of *Lactobacillus plantarum* 23. The biomass was obtained after 8 days of incubation in 1-L outdoor PBR under 6% CO_2_ condition. Data are presented as mean ± SD (*n* = 3)

* Following the SD indicates significant difference at *P* < 0.05 using student *t* test

**Table 3:** The time-course lactic acid production with *Lactobacillus plantarum* 23 and the consumption of glucose and xylose from hydrolysate of microalgal feedstock

Time (h)	Control	WT^a^		M4^b^		Lactic acid^c^ (g/L)		
	Glucose (g/L)	Glucose (g/L)	Xylose (g/L)	Glucose (g/L)	Xylose (g/L)	Control	WT	M4 mutant
0	20.5 ± 0.32	21.0 ± 0.33	1.9 ± 0.06	21.4 ± 0.29	2.1 ± 0.10	0	0	0
3	4.5 ± 0.17	2.65 ± 0.08	0.21 ± 0.00	2.75 ± 0.13	0.2 ± 0.01	11.60 ± 0.36	13.10 ± 0.54	13.45 ± 0.39
5	0	0	0	0	0	14.50 ± 0.28	16.10 ± 0.40	16.38 ± 0.62
8	0	0	0	0	0	14.40 ± 0.12	16.13 ± 0.08	16.45 ± 0.13
12	0	0	0	0	0	14.50 ± 0.12	16.13 ± 0.08	16.45 ± 0.13
24	0	0	0	0	0	14.50 ± 0.12	16.13 ± 0.08	16.45 ± 0.13
Conversion rate^d^ (%)						74.87	80.42	79.17

The WT and M4 mutant feedstock was obtained after 8 days of culture under 6% CO_2_ condition. Pure glucose (used as control) in the fermentation broth of the control test was 20.5 g/L and accordingly, the content of glucose in the hydrolysate of the wild type (WT) and M4 mutant in the fermentation broth was adjusted to around 21 g/L with a xylose content of around 2 g/L. The conversion rate is estimated as (lactic acid content/theoretical lactic acid content) × 100 after 24 h of fermentation. The data are presented as mean ± SD (*n* = 3)

^a^47.15 g day wt. of WT feedstock in 1 L fermentative mixture

^b^39.28 g day wt. of M4 feedstock in 1 L fermentative mixture

^c^Lactic acid in 1 L fermentative mixture

^d^(Real lactic acid content/theoretical lactic acid content) × 100

## Discussion

Overheating of PBR in outdoor condition is one of the major problems that limits the growth of algal cells. It could be overcome via the efficient temperature control in outdoor PBRs [24]. Besides, the selection of heat-tolerant species provides an alternative solution. Here we have successfully selected a heat-tolerant mutant (M4) from *Chlorella* sp. GD after MNNG mutagenesis. M4 exhibited high biomass productivity of 1.01 g/day/L in outdoor PBR, in which the temperatures can reach up to 49 °C at noon. This is not only higher than other *Chlorella* spp. [25, 26] but also exceed other green algae, for example, 0.840 g/day/L for *Scenedesmus* sp. CNWN [27] and 0.796 g/day/L for *Desmodesmus* sp. [28]. It is also comparable to the biomass productivity of mixotrophically cultured *Chlorella sorokiniana* in the presence of 2% (w/v) glucose, 1.178 g/L/day [12]. It is obvious that this heat-tolerant mutant M4 is a promising strain for outdoor culture of microalgae in southern Taiwan under hot weather.

*Chlorella* mutant M4 is a potential fermentative feedstock for large-scale lactic acid production. The results obtained from indoor and outdoor PBRs illustrate that M4 adapts to high-temperature environment and high-intensity illumination in outdoor condition when aerated with 6% CO_2_ and its biomass production can reach up to 8.22 g/L. The total reducing sugar content after H_2_SO_4_ hydrolysis was 59.83% with glucose and xylose as the major components. It is therefore a suitable feedstock for the production of lactic acid through fermentation. Using a lactic acid-producing bacterium *L. plantarum* 23, the lactic acid conversion rate of M4 biomass and the lactic acid yield were 79.17% and 0.43 g/g, respectively. Our findings agree with the notion that algae are a promising biomass species due to their high CO_2_ fixation ability than higher plants and a feedstock for fermentative production of many chemicals such as lactic acid due to their abundant polysaccharides, proteins, phosphate, vitamins, and minerals [29, 30].

The fermentative conversion rate of M4 biomass into lactic acid (79.17%) was higher than that of glucose (74.87%). This can be attributed to the presence of xylose. The existence of organic nitrogen (amino acid and peptides), vitamins, and other nutrients in acid hydrolysate may also contribute to higher lactic acid yield of algal biomass. Although we did not determine these constituents in the acid hydrolysate of *Chlorella* biomass, the nitrogen content in algal biomass was 5–7%, and it can be utilized as the nitrogen nutrient for the lactic acid-producing bacterium *L. plantarum* 23. It is known that supplementation of nitrogen [31] and/or vitamin B [32] alone or together enhanced lactic acid production from *Lactobacilli*. It is expected that the nitrogen-containing compounds and vitamins in algal biomass augment lactic acid synthesis in the bacterium *L*. *plantarum* 23 during growth and fermentation. Yeast extract is generally used as the source of amino acids and vitamin B for lactic acid fermentation [33], but it is the major contributor of medium cost for industrial lactic acid production [34]. Using dry biomass of a freshwater green microalga, *H*. *reticulum*, the amount of yeast extract and peptone for lactic acid fermentation has been markedly reduced [13]. The potential of *Chlorella* biomass in the replacement of yeast extract for lactic acid production will be determined in the near future.

Although PSII activity (*F*_v_′/*F*_m_′) was similar between wild type and the mutant M4 after 3–4 days of culture, the photosynthetic O_2_ evolution rate was higher in M4 mutant compared to the wild type (Fig. 4). This is because the PSII activity is the representation of mainly PSII function while the photosynthetic O_2_ evolution rate is the combination of PSII activity, electron transport rate, and Calvin cycle function. Therefore, the data obtained indicate that the photosynthetic electron transport rate and the function of Calvin cycle could also be enhanced in this mutant M4. It is worth noting that since the determination of photosynthetic O_2_ evolution rate was carried out at 25 °C as described in the “Methods” section, the photosynthetic ability of the microalgae was assessed after temperature and light treatment with and without the CO_2_ supplement.

The photosynthetic CO_2_ fixation and light utilization efficiency have been modified in the *Chlorella* sp. GD following the gain of enhanced thermo-tolerance. Although the DIC affinity and the initial slope (α) of PI curve were similar between wild type and the mutant, the DIC-dependent photosynthetic O_2_ evolution rate (i.e., the capacity of DIC utilization) and the value of *P*_max_ were greatly enhanced in the mutant grown under 40 °C, 200 μmol/m^2^/s, and 6% CO_2_ condition. It is therefore proposed that the genotypic alterations observed in mutant strains lead to enhanced carbon reduction through the Calvin cycle, its subsequent flux into sugars and their immobilization in starch, although the transport of CO_2_ and/or HCO_3_^−^ through cell membrane is not modified in M4. Furthermore, a similar initial slope (α) of PI curve and the elevation of *P*_max_ value reflect that M4 possesses enlarged light trap (photosystems number) but fixed antenna size for the absorption of light energy when light intensity is increased. Such potential genetic modifications in photosynthetic functions lead to high biomass and sugar production in M4 cultured in outdoor PBR aerated with enriched CO_2_. Therefore, M4 is a potential candidate for photoautotrophic mitigation of CO_2_ from flue gas in outdoor conditions.

The photosynthetic O_2_ evolution rate (PI curve) shown in Fig. 5A–D also demonstrates that the photosynthetic O_2_ evolution rate of M4 mutant was higher than that of the wild type in response to light intensity ≥200 μmol/m^2^/s, especially under the supply of 6% CO_2_. Therefore, 200 μmol/m^2^/s was selected as high light intensity in the indoor PBR study. The biomass production was not examined in the light intensity higher than 200 μmol/m^2^/s in the indoor PBR experiments. However, the biomass production of M4 mutant in the field test (light intensity can reach up to around 1400–1800 μmol/m^2^/s at noon) was found to be significantly higher than that of the wild type. The enhancement of photosynthesis and biomass production in this heat-tolerant mutant M4 by enriched CO_2_ is dependent on elevated light intensity and temperature. Because CO_2_ fixation by algae is a light-dependent process, illumination at low light intensity (50 μmol/m^2^/s) does not provide enough photons for the light-dependent reactions in M4 to provide sufficient energy for CO_2_ fixation under 6% CO_2_ and 40 °C condition. Further, the reaction rate in Calvin cycle can be accelerated by elevated temperatures. In indoor PBR, the combination of high temperature (40 °C), elevated light intensity (200 μmol/m^2^/s), and enriched CO_2_ (6%) is required for the maximal biomass production. A similar result has been observed in the previous study using *C*. *vulgaris* ARC1 to examine the interactive effect of CO_2_ and temperature on algal biomass production in 47 μmol/m^2^/s condition; the highest biomass occurred at 6% CO_2_ and elevated temperature (30 °C) [35]. It is recognized that CO_2_, light, and temperature, are the limiting factors for photosynthesis [36–38]. These factors interact in the regulation of photosynthetic activity [37, 39, 40]. Therefore, promotion of photoautotrophic biomass production of the mutant M4 can be attributed to enhanced photosynthesis with CO_2_ as inorganic carbon source under elevated temperature and high light intensity.

The mechanism for the mutant with enhanced high-temperature tolerance is likely involved in the mutation of the genes associated with heat shock proteins and their related components. In response to the increase in temperature tolerance, the genes involved in enlarged PSII number (such as light harvesting complex), as reflected by the elevation of *P*_max_ value, and enhanced CO_2_ fixation process (the function of CO_2_ utilization and Calvin cycle), as reflected by increased DIC-dependent photosynthetic O_2_ evolution rate, are also mutated. Alternatively, it is also likely that photosynthesis can be enhanced in M4 mutant because heat shock proteins could protect photosynthetic electron transport during stress [41].

## Conclusions

The mutant strain of *Chlorella* sp. GD with enhanced thermo-tolerance and higher CO_2_ fixation ability shows higher biomass productivity of 8.22 g/L in hot outdoor PBR. The photosynthetic CO_2_ fixation and light utilization efficiency have been modified in the *Chlorella* mutant. This ability makes M4 a potential candidate for the mitigation of CO_2_ using outdoor PBR. The mutant composed of higher reducing sugar is a suitable feedstock for fermentative lactic acid production with a yield of 0.43 g/g. This heat-tolerant mutant is a promising microalgal species and a potential feedstock for fermentative lactic acid production. However, the feasibility for cost-competitive lactate production from algal carbohydrates produced with photobioreactors (PBRs) is challenged by the high cost associated with PBR operations. In our future work, open pond system will be used to cultivate mutant M4 to produce algal biomass with high carbohydrate content in a cheaper way to further evaluate the economic feasibility of lactic acid production using the microalgal biomass as feedstock.

## Methods

### Algal strain and mutation

The stock cells of *Chlorella* sp. GD were phototrophically and axenically cultured in 50 mL BG-11 medium (pH 8.0, NaNO_3_ as adjusted to 1 g/L) [42] in a 125 mL flask (PYREX, Germany) and agitated on an orbital shaking incubator (model OS701, TKS company, Taipei, Taiwan) at 150 rpm under continuous illumination with white light [50 μmol/m^2^/s measured by a LI-250 Light Meter with a LI-200SA pyranometer sensor (LI-COR, Inc., Lincoln, Nebraska, USA)] at 28 °C. When cell density determined by OD_682_ reached 0.4–0.5, 15 mL of culture (2×10^6^ cells/mL) was centrifuged at 4000×*g* for 3 min and subject to chemical mutagenesis by treating with 0.05 mg/mL *N*-methyl-*N*′-nitro-*N*-nitrosoguanidine (MNNG, filtrated through 0.22 μm filter) for 1 h. According to the survival curve for mutagenesis with MNNG, the MNNG concentration of 0.05 mg/mL that resulted in around 5% of cell viability (data not shown) was chosen in this study. After removal of MNNG solution by centrifugation at 4000×*g* for 3 min and then again washing with fresh 15 mL BG-11 medium, the cells were suspended in 1 mL BG-11 medium with shaking (100 rpm) for recovery under low light intensity of 5–10 μmol/m^2^/s at 25 °C for 24 h. Then, algal cells were spread on the agar (BG-11) with 0.2 mL for one plate and these five plates were incubated at 40 °C and 100 μmol/m^2^/s illumination for high-temperature tolerance screening. After 1 week, 179 colonies appeared on the plate and was transferred to axenic test tube containing 5 mL BG-11 and incubated in a shaker incubator at 40 °C and 200 μmol/m^2^/s illumination for further screen. Thirty-one colonies became green after 3 days which were then transferred to 125 mL flask containing 50 mL BG-11 for a second round of screening. Two of the colonies which exhibited a higher growth rate compared to wild type were obtained and the best performing mutant M4 was chosen for further studies.

In the attempt to check whether this mutant can tolerate high-temperature stress in the field conditions in southern Taiwan in the tropical region where air temperatures could reach around 35 °C and water temperatures would surpass 40–45 °C during the daytime in the summer, a 1-L vertical column glass PBR (60 cm height and 5 cm internal diameter) with gas inlet at the bottom was set up in outdoor conditions without temperature control. The medium in PBR was aerated with ambient air or 6% CO_2_ at 0.2 vvm (air volume/culture volume/min). The medium in PBR was stirred by magnetic stirrer with a speed of 100 rpm. Both air temperatures and irradiance outside the PBR and water temperatures and irradiance in the center of the PBR were recorded for evaluation of thermal tolerance and enhanced light tolerance of M4. The initial cell density for cultivation was set at OD_682_ = 0.4. For reducing sugar production to be used as a feedstock for lactic acid fermentation, the algal cells after 8 days of culture in outdoor PBR were harvested after centrifugation at 10,000×*g* and lyophilized at −50 °C in a lyophilizer. The dried biomass was stored at −20 °C until analysis.

The effects of temperature, CO_2_, and light intensity on biomass and photosynthetic efficiency were studied using small PBR setup in the laboratory. A 75-mL vertical column glass PBR (10 cm height and 5 cm internal diameter) with gas inlet at the bottom was set up. Ambient air or CO_2_ in different concentrations (2, 6, and 20%) was used for aerating the culture at the rate of 0.5 vvm. The temperature was controlled at 25, 40, or 48 °C and light intensity was set up at 50 or 200 μmol/m^2^/s with an external light source (14 W TL5 tungsten filament lamps, Philips Co., Taipei, Taiwan) mounted on both sides of the PBR for 24 h illumination. The cell density was determined by the absorbance at 682 nm. The initial cell density for culture in PBR was set at OD_682_ = 0.4 (~0.16 g dry weight/L).

### Algal biomass estimation

The biomass of *Chlorella* sp. GD wild type and mutant M4 was determined using UV/VIS spectrophotometer via the measurement of optical density at a wavelength of 682 nm (OD_682_). The absorbance values were converted to dry weight via appropriate calibration (1 OD_682_ = 0.410 g/L). The initial biomass for indoor PBR culture and outdoor OBR culture was ~0.16 g dry weight/L.

### Determination of photosynthetic O_2_ evolution rate

The amount of O_2_ released or uptake by algal cells were detected using a Clark-type oxygen electrode fitted with a DW3 chamber (Hansatech, Kings Lynn, Norfolk, England) with temperature controlled at 25 °C using a thermostat. For sampling, the cells were collected and centrifuged at 5000×*g* for 10 min and then the cell pellet was suspended in 50 mM HEPES buffer (pH 7.4) with a density of 1.0 × 10^6^ cells/mL containing 5 mM NaHCO_3_. Then, the photosynthetic O_2_ evolution was determined at a light intensity of 800 μmol/m^2^/s till stable slope appeared. After that, light was turned off to determine respiratory O_2_ uptake rate. Because the preliminary test showed that photosynthetic O_2_ evolution reached plateau during 600–1400 μmol/m^2^/s, followed by a slight drop during 1600–2000 μmol/m^2^/s, the intensity of 800 μmol/m^2^/s was chosen for the determination of photosynthetic O_2_ evolution rate. The light source used was low voltage Tungsten halogen lamps (12 V, 50 W; Sylvania, Danvers, MA, USA). Three replicates per treatment were performed. The net photosynthetic O_2_ evolution rate and the respiration rate were expressed as μmol O_2_ evolution/h/10^6^ cell and μmol O_2_ uptake/h/10^6^ cell, respectively. The gross photosynthetic O_2_ evolution rate was the sum of net photosynthetic O_2_ evolution rate and the respiration rate.

### Chlorophyll *a* fluorescence determination of PSII activity using pulse amplitude modulation (PAM) fluorometry

Chlorophyll *a* fluorescence parameters were employed to determine the activity of photosystem II (PSII) using AP-C 100 (AquaPen, Brno, Czech Republic). A 0.5-mL aliquot of an algal culture was diluted with TAP medium to an OD_682_ = 0.10–0.15 with a chlorophyll *a* content of 1.5–2.2 μg/mL. A 2-mL aliquot of diluted algal cells was then transferred to an AquaPen cuvette and subjected to a pulse of saturating light of 4000 μmol/m^2^/s photosynthetically active radiation (PAR) to obtain the light-adapted minimal fluorescence (*F*_t_) and the light-adapted maximal fluorescence (*F*_m_’). To determine the maximum PSII activity, *F*_v_/*F*_m_ (=*F*_m_ − *F*_o_/*F*_m_), 2 mL of diluted algal cells in an AquaPen cuvette was incubated in the dark for 30 min and then flashed with saturated light (4000 μmol/photons/m^2^/s) to obtain the dark-adapted minimal fluorescence (*F*_o_) and dark-adapted maximal fluorescence (*F*_m_). The active PSII activity, *F*_v_’/*F*_m_’ = *F*_m_’ − *F*_t_/*F*_m_’, and the maximum PSII activity, *F*_v_/*F*_m_ = *F*_m_ − *F*_o_/*F*_m_, were then calculated.

### Determination of carbohydrate, nitrogen, and sugar content

For the determination of algal tissue C and N contents, 100 mL of cells was centrifuged at 12,000×*g* for 5 min at 4 °C and then the pellet was lyophilized at −50 °C. The dry algal sample of 0.01 g was used for the determination of C and N by the Elementar vario EL III CHNOS Elemental Analyzer (Elementar, Hanau, Germany).

The carbohydrate composition in wild type and mutant was determined using the modified quantitative saccharification (QS) method reported by the Nation Renewable Energy Laboratory (NREL), USA [43]. A small amount of dry cell powder (~0.1 g dry weight) was added to 3 mL of 72% (w/w) sulfuric acid and incubated for 60 min at 30 °C for the primary hydrolysis. The hydrolysate was then diluted to 4% (w/w) sulfuric acid and incubated for 20 min at 121 °C (sterilization) as the secondary hydrolysis. The supernatant was neutralized and analyzed by high performance liquid chromatography for the concentrations of reducing sugars present and the total carbohydrates obtained.

### Acid hydrolysis of microalgal biomass

Lyophilized microalgae powder (40 g/L) was mixed with sulfuric acid at a final concentration of 4% (w/w). The slurry was autoclaved for 20 min at 121 °C. After acid hydrolysis, the hydrolysate was cooled to room temperature and then centrifuged at 25 °C and 10,000 rpm for 5 min. After cooling to room temperature, microalgae hydrolysate was neutralized with CaCO_3_ and the formed solid precipitate was removed by centrifuging at 13,500 rpm for 5 min. The supernatant containing the released sugars was collected as acid hydrolysate, whose sugar content was measured with a high performance liquid chromatograph machine equipped with a refraction index detector (RID-10A, Shimadzu, Japan) [44]. HH was prepared as previously described [45]. In all experiments, the hydrolysate was supplemented with all other components of the modified MRS media (see the next section) except for the primary carbon source. A concentrated solution containing the modified MRS components was autoclaved at 121 °C for 20 min and added to the treated hydrolysate.

### Production of lactic acid by separate hydrolysis and fermentation (SHF)

The efficiency of the conversion of hydrolyzed microalgal biomass into lactic acid was examined with the lactic acid-producing bacterium *Lactobacillus plantarum* 23. After acid hydrolysis of the microalgal biomass, following the procedures described above, the residue was removed via centrifugation (10,000 rpm, 5 min) and the pH of hydrolysate was adjusted to 5.5 with NaOH to be suitable for lactic acid fermentation with *L. plantarum* 23. Fermentations were carried out under anaerobic conditions with 100 mL modified MRS medium in reactor. The culture conditions were maintained at pH 5.5, temperature 30 °C, and 200 rpm agitation for 24 h. The modified MRS medium was composed of 10 g/L peptone, 10 g/L beef extract, 1 mL/L Tween 80, 2 g/L K_2_HPO_4_, 5 g/L CH_3_COONa, 2 g/L ammonium citrate, 0.1 g/L MgSO_4_∙7H_2_O, 0.05 g/L MnSO_4_∙5H_2_O. For carbon source, 20 g/L glucose or acid hydrolysate equivalent to 20 g/L glucose was added in the modified MRS medium.

### Statistics

All experiments were repeated at least three times and because they showed a similar trend, only the results for one of them are shown in this paper. Statistical analyses were performed using SPSS (SPSS 15.0 for Windows Evaluation Version). Significant differences between sample means were analyzed using Student’s *t* test or Duncan’s new multiple range test at *P* < 0.05.

## Abbreviations

DIC: dissolved inorganic carbon
MNNG: *N*′-nitro-*N′-*nitro-*N*-nitrosoguanidine
OD: optical density
PBR: photobioreactor
PAM: pulse amplitude modulation
PAR: photosynthetically active radiation
PI curve: the photosynthetic light response curve
*P*_max_: the maximum photosynthesis rate
QS: quantitative saccharification
SHF: separate hydrolysis and fermentation
vvm: air volume/culture volume/min
